# Influence of Fe Vacancy on the Bonding Properties of γ-Fe (111)/α-Al_2_O_3_ (0001) Interfaces: A Theoretical Study

**DOI:** 10.3390/ma18204666

**Published:** 2025-10-11

**Authors:** Xiaofeng Zhang, Renwei Li, Qicheng Chen, Dehao Kong, Haifeng Yang

**Affiliations:** 1 College of Mechanical and Electrical Engineering, Jilin Institute of Chemical Technology, Jilin 132022, China; 2 School of Energy and Power Engineering, Northeast Electric Power University, Jilin 132012, China; 3 College of New Energy and Materials, Northeast Petroleum University, Daqing 163711, China; 4 State Key Laboratory of Advanced Welding and Joining, Harbin Institute of Technology, Harbin 150001, China

**Keywords:** ceramics/metal interface, bonding strength, electronic properties

## Abstract

Here, the effects of Fe vacancy defects on the bonding properties of γ-Fe (111)/α-Al_2_O_3_ (0001) interfaces are studied in depth at the atomic and electronic levels using first-principles calculations. The first (V_1_), second (V_2_), third (V_3_), and fourth (V_4_) layers of vacancy structures within the Fe substrate, as well as the ideal Fe/Al_2_O_3_ interface structure, are proposed and contrasted, including their thermodynamic parameters and atomic/electronic properties. The results demonstrate that the presence of vacancies in the first atomic layer of Fe deteriorates the interfacial bonding strength, whereas vacancies situated in the third layer enhance the interfacial bonding strength. The effect of vacancy beyond the third layer becomes negligible. This occurs mainly because vacancy defects at different positions induce the relaxation behavior of atoms, resulting in bond-breaking and bond-forming reactions at the interface. Following that, the formation process of vacancies can cause the transfer and rearrangement of the electrons at the interface. This process leads to significant changes in the charge concentration of the interfaces, where V_3_ is the largest and V_1_ is the smallest, indicating that the greater the charge concentration, the stronger the bonding strength of the interface. Furthermore, it is discovered that vacancy defects can induce new electronic orbital hybridization between Fe and O at the interface, which is the fundamental reason for changes in the properties of the interface. Interestingly, it is also found that more electronic orbital hybridization will strengthen the bonding performance of the interface. It seems, then, that the existence of vacancy defects not only changes the electronic environment of the Fe/Al_2_O_3_ interface but also directly affects the bonding properties of the interface.

## 1. Introduction

Metal matrix ceramic (Fe-Al_2_O_3_) composites synergistically combine the toughness and strength of metal with the hardness, thermal stability, and corrosion resistance of ceramic phases, enabling widespread applications in critical sectors, including energy, aerospace, automotive, and biomedical engineering [1,2,3,4,5]. However, the poor wettability between Fe and Al_2_O_3_ has become a critical limiting factor for their long-term applications and technological development. Therefore, it is imperative to investigate the microscopic properties of Fe/Al_2_O_3_ interfaces, from atomic bonding configurations to electronic interactions.

In recent years, extensive efforts have been made to optimize Fe/Al_2_O_3_ interfaces through various fabrication methods, including co-precipitation [6], self-propagation high-temperature synthesis [7], hydrothermal synthesis [8], the sol–gel method [9], and mechanical alloying [10]. Of particular interest, references [11,12] revealed that nano- Al_2_O_3_ can spontaneously adsorb onto Fe surfaces without requiring additional processing, subsequently forming a protective film. These investigations consistently identified pore and cavity formation at Fe/Al_2_O_3_ interfaces, manifesting as vacancy-type defects. Such defects may significantly affect the stability of the interfacial bonding, thereby modulating the macroscopic properties of the material. Therefore, elucidating the structure property relationship between vacancy defects and material degradation mechanisms is of crucial scientific and practical importance.

The first-principle calculations are, by far, one of the most frequently employed methods in the study of solid–solid interface theory due to their basis in quantum mechanics [13,14]. Some scholars have studied the properties of metal/Al_2_O_3_ interfaces from the atomic and electronic level by first-principle calculations. Chen et al. [15] investigated various NiTi (111)//α-Al_2_O_3_ (0001) interface configurations and found that the Ti-O-MT and Ni-O-MT configurations were the most stable among all interface models. Hocker et al. [16] studied the most stable M (M = Ag, Cu, Nb, Al)/Al_2_O_3_ interface structure and revealed the fracture mechanism of the interface. Additionally, it was found that the bonding strength of the interface is directly related to the interface spacing between M and Al_2_O_3_. Shi et al. [17] demonstrated that the O-terminated configuration of the Ni (111)/Al_2_O_3_ (0001) interface showed the highest bonding strength in tensile tests. Alternatively, the failure position of the O-terminated Ni/Al_2_O_3_ structure did not occur at the interface during the tensile process, but inside the Ni block. Xie et al. [18] introduced Ti and Ni to the Fe/Al_2_O_3_ interface and found that Ti significantly enhances the interfacial bonding strength, while Ni may deteriorate it. Peng et al. [19] investigated vacancy defects at the Fe/Y_2_O_3_ interface and found that vacancies are more likely to form on the Fe matrix side, with mono-vacancies exhibiting a relatively low formation energy. Some of these monovacancy can effectively enhance the material’s properties. In recent studies, our group has conducted in-depth research on the bonding properties, failure process, and strengthening mechanism of the Fe/Al_2_O_3_ interface under ideal conditions [20,21,22,23]. The results are as follows: (1) The Fe/Al_2_O_3_ interface structure with O-termination is found to have the best performance. (2) Suitable rare earth elements (Ce, Y, Sc, La, Er, Yb, Gb, and Nd) and active metal elements (Ti and Mg) can strengthen the bonding properties of the Fe/Al_2_O_3_ interface. Therefore, first-principles calculations based on quantum mechanics can effectively predict the formation process of metal–ceramic interfaces and characterize their properties, providing a theoretical foundation for experimental investigations.

For the novel Fe-Al_2_O_3_ composite fabrication method, the formation process of the Fe/Al_2_O_3_ interface involves complex mechanisms, including nanoparticle adsorption, deposition, and bonding, while simultaneously generating vacancy defects at the interfaces. Such defects may originate from oxygen vacancies on the alumina side or iron vacancies on the Fe side. Relevant studies [19,24] indicate that the formation energy of vacancy defects on the metal substrate side is relatively low, which may facilitate their spontaneous formation under certain conditions.

Based on the above discussion, this paper will explore the effect of single vacancy defects at the first, second, third, and fourth layers of the Fe substrate on the bonding properties of the Fe (111)/Al_2_O_3_ (0001) interface at the atomic and electronic levels. This work consists of main steps, detailed below. First, ideal and non-ideal Fe/Al_2_O_3_ interface structures with different defect positions are created to achieve effective contrast. Second, the relationship between vacancy defects and interfacial bonding is discussed by calculating work of adhesion, vacancy formation energy, and atomic relaxation behavior. Last, the electronic properties of the Fe/Al_2_O_3_ interface in the defect state, including charge density, charge density difference, and partial density of states, etc., are calculated to obtain the influence of vacancy defects on the bonding mechanism of interface. Our findings provide valuable insights into the practical applications.

## 2. Calculation Method and Details

### 2.1. Calculation Settings

All calculations are performed using the Cambridge Sequence Total Energy Package (CASTEP) code [25]. First, this work uses the density functional theory framework and uses the Perdew–Burke–Ernzerhof (PBE) function under the generalized gradient approximation (GGA) method to describe the exchange–correlation potential energy [26,27]. Second, the energy convergence test is carried out on the kinetic energy cutoff value, and the plane-wave cutoff is set to 380 eV. The Brillouin zone uses Monkhorst–Park k-point grid sampling. The two block k-point grids are set to 8 × 8 × 8 with high precision, and the k-point grids of interface models are set to 8 × 8 × 1. The O-2s^2^2p^4^, Al-3s^2^3p^1^, and Fe-3d^6^4s^2^ are selected as valence electrons of atoms. Last, the Broyden–Fletcher–Goldfarb–Shannon (BFGS) [28] algorithm is utilized to optimize the block, surface, and interface models. In terms of computational precision, the convergence threshold of the energy change in the relaxation process is set to 1.0 × 10^−5^ eV/atom, maximum displacement in the optimization process is 0.001 Å, and average atomic force is reduced to 0.03 eV/Å.

### 2.2. Model Building

**Figure 1:**
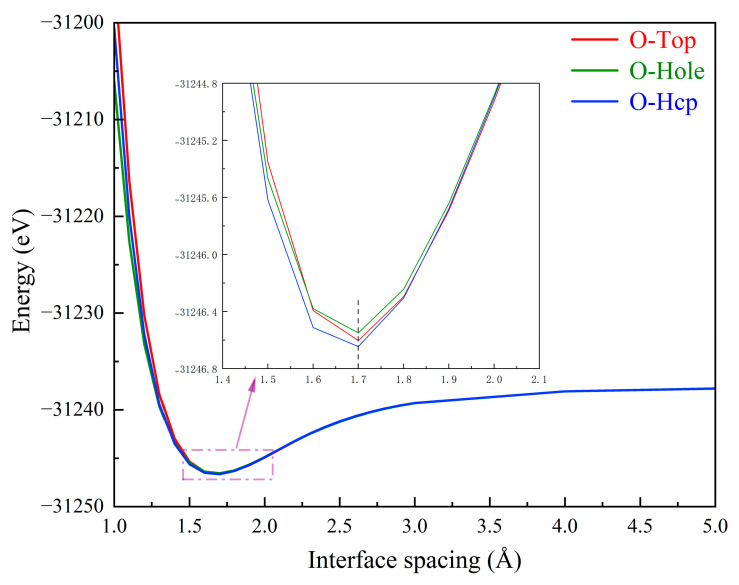
The relationship between interface spacing and structural energy.

According to the interface characteristics of heterogeneous materials [29], the γ-Fe (111)/α-Al_2_O_3_ (0001) interface, which exhibits a low lattice mismatch of 0.116%, is chosen. According to previous work from our research group [20], using 7 atomic layers for Fe and 12 atomic layers for Al_2_O_3_ bulk structures ensures computational accuracy. The O-terminated γ-Fe (111)/α-Al_2_O_3_ (0001) interface is the most stable among the various structures and is therefore chosen to be the basic model for this work. Then, the optimal interface spacing is determined through screening, as shown in Figure 1, and finally the O-terminated interface with a spacing of 1.7 Å at the Hcp site is selected as the research model. After relaxation, the structural interface spacing is reduced to 1.16 Å.

**Figure 2:**
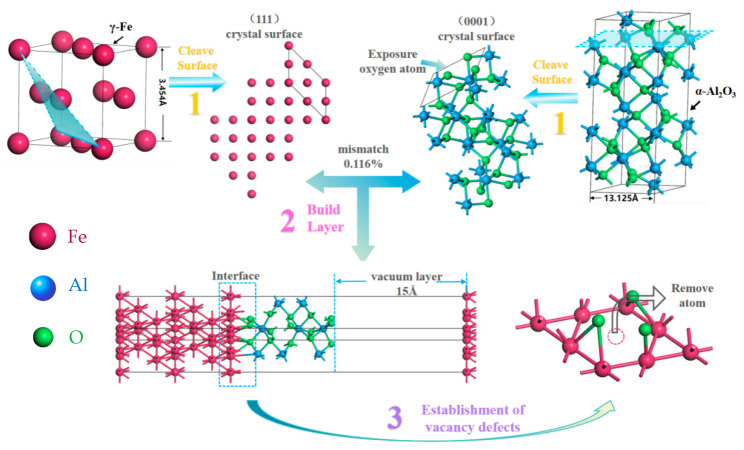
The establishment process of Fe (111)/Al_2_O_3_ (0001) interface configuration.

**Figure 3:**
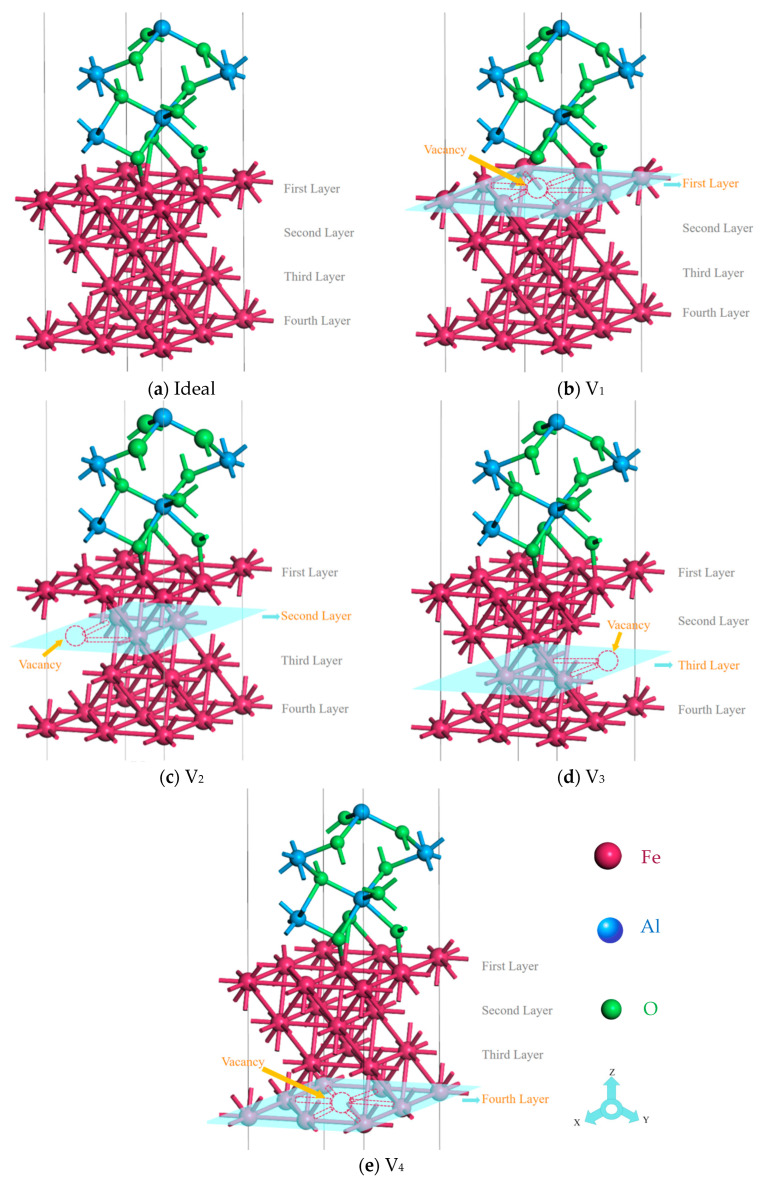
The ideal and defective Fe/Al_2_O_3_ interface structures.

In order to eliminate the influence of periodic boundary conditions, it is necessary to add a vacuum layer in the z-direction of the Fe/Al_2_O_3_ interface model [30]. Here, the thickness of the vacuum layer is selected to be 15 Å. Additionally, the upper and lower surfaces of Fe and Al_2_O_3_ are set as symmetrical atomic structures to eliminate the dipole moment effect [31]. Thus, the Fe surface with 7-layer atoms and the Al_2_O_3_ surface with 12-layer atoms are used to constitute the Fe/Al_2_O_3_ interface. The construction process of the γ-Fe (111)/α-Al_2_O_3_ (0001) interface model is illustrated in Figure 2 (red represents Fe, green represents O, and blue represents Al). Motivated by this, here we fix the vacancy concentration (a single Fe vacancy, concentration 25%), and the position of vacancy is set on the first, second, third, and fourth layers of Fe atoms (hereafter abbreviated as V_1_, V_2_, V_3_, and V_4_, respectively), as shown in Figure 3 (the model in the figure represents the actual supercell used in the calculations).

## 3. Results and Discussion

### 3.1. Bonding Strength of the Interface

As an important thermodynamic parameter, the work of adhesion (hereafter referred to as W_ad_) is useful for assessing the bonding strength of the solid/solid interface. As a rule, the larger the value of W_ad_, the stronger the binding strength of the interface. To explore the effects of vacancy defects on the Fe/Al_2_O_3_ interface, it is necessary to calculate the W_ad_ of ideal and non-ideal Fe/Al_2_O_3_ interface structures. Herein, the W_ad_ is calculated by Equation (1) [32]:

(1) $$W_{ad}=\frac{E_{Fe}+E_{{\mathrm{Al}}_{2}O_{3}}-E_{Fe/{\mathrm{Al}}_{2}O_{3}}}{A}$$

where E_Fe_ and $E_{{\mathrm{Al}}_{2}O_{3}}$ represent the energy of the Fe-only block and the Al_2_O_3_-only block, respectively, when the other sides of the model is replaced by a vacuum layer. $E_{Fe/{\mathrm{Al}}_{2}O_{3}}$ is the total energy of the Fe/Al_2_O_3_ interface structure and A is the area of the interface.

**Figure 4:**
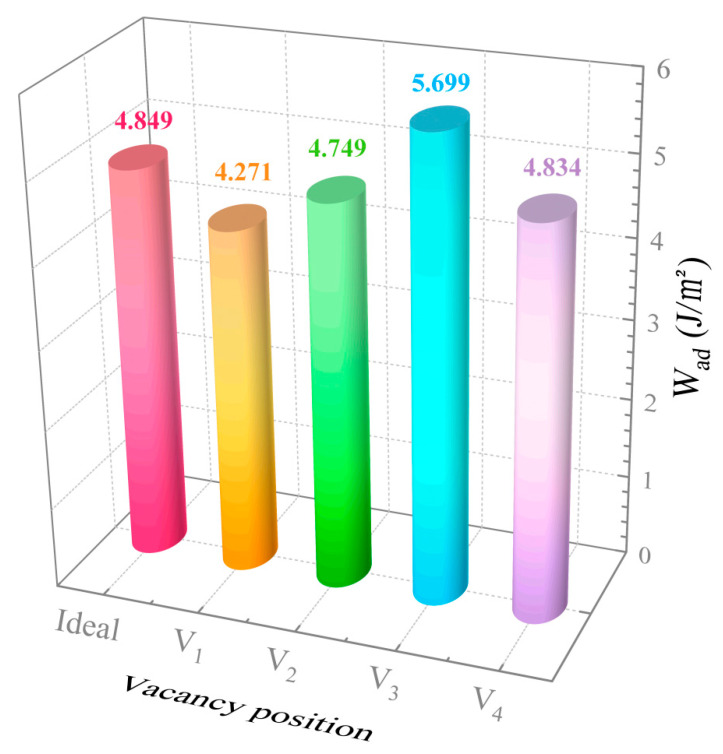
The work of adhesion of ideal and defective Fe/Al_2_O_3_ interface structures.

Figure 4 displays the W_ad_ of ideal and defective Fe/Al_2_O_3_ interface structures. Relative to the ideal Al_2_O_3_/Fe interface, the vacancy at the first layer of the V_1_ structure leads to a 12% decrease in the bonding strength of the interface. It seems, then, that reducing the vacancy defects at the outermost atomic layer of matrix Fe is an effective measure to improve the service life of the Fe/Al_2_O_3_ composite. However, the W_ad_ increases by 17.5% compared to the ideal Fe/Al_2_O_3_ interface when the vacancy defect is located in the third layer, referred to as V_3_. This is most likely to be due to the fact that vacancy defects induce the lattice distortion of the local structure and promote the toughening of the interfacial region. Moreover, the V_2_ and V_4_ structures have little effect on the binding of the Fe/Al_2_O_3_ interface. It is shown, thus, that the effect of vacancies at different positions on Fe/Al_2_O_3_ interface may be positive, negative, or neutral. Relevant studies [33,34] have also shown that the effect of defects on the performances of interface may be positive or negative, which matches our research.

To better understand the effect of vacancy defects on the bonding strength of Al_2_O_3_/Fe interface, the vacancy formation energy is further calculated and atomic migration behavior at the interface is observed. The vacancy formation energy (E_f_) helps to understand the generation and evolution mechanisms of defects in materials. Specifically, a lower E_f_ indicates that less energy is required to form a vacancy defect, making it easier to form and more stable in the crystal. Conversely, a higher E_f_ means that more energy is needed to form a vacancy defect, making it harder to form and less stable in the crystal. It can be obtained by the following Equation (2) [35]:

(2) $$E_{f}=E_{V}-E_{Fe/{\mathrm{Al}}_{2}O_{3}}+nE_{atom}$$

where E_V_ represents the total energy of the structure with defects, $E_{Fe/{\mathrm{Al}}_{2}O_{3}}$ represents the total energy of the ideal Fe/Al_2_O_3_ interface structure, n is the number of additional or missing atoms, and E_atom_ denotes the energy of the additional or missing atoms.

**Figure 5:**
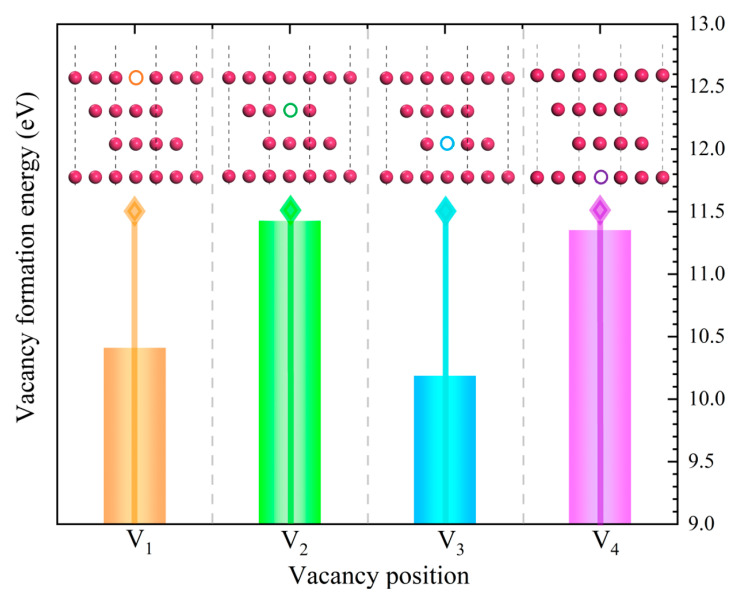
Formation energy of vacancy for defective Fe/Al_2_O_3_ interface structures.

Figure 5 is the vacancy formation energy of the Fe/Al_2_O_3_ interface structure with different positions of vacancy. It can be seen that the E_f_ of all structures is positive, indicating that defects need to consume energy to form and can exist in the Fe/Al_2_O_3_ interface structure [36]. Significantly, the E_f_ of the V_1_ and V_3_ structures is relatively small, indicating that vacancy defects are more likely to form at these positions of the Fe/Al_2_O_3_ interface and are stabilized within the structure. On the contrary, vacancies in V_2_ and V_4_ structures are unstable and even disappear through atom diffusion or other mechanisms. Based on the results of W_ad_ and E_f_, it can be inferred that the more stable the vacancies at or near the interface, the stronger the interfacial bonding. Among them, the vacancy formation energy of V_3_ is the smallest, indicating that the structure is the most stable, i.e., the bonding strength of interface is the highest, consistent with the results of W_ad_ above. In other words, the structural stability is influenced by whether vacancy defects can exist in a stable manner.

**Figure 6:**
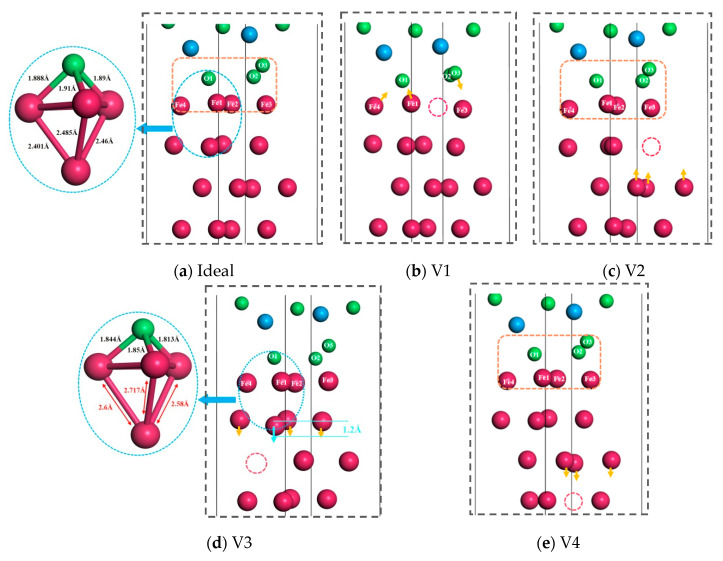
Local structure of ideal and defective Fe/Al_2_O_3_ interfaces after relaxation.

The formation of vacancy defects will inevitably be accompanied by lattice distortion, and the atoms of interface will be rearranged to achieve a stable state. Consequently, it is necessary to analyze the interface structures after optimization. Figure 6 shows the local structure of the ideal and non-ideal Fe/Al_2_O_3_ interfaces after relaxation. For V_2_ and V_4_ structures, the movement of atoms is very small compared to the ideal Fe/Al_2_O_3_ interface, which has little effect on the bonding strength of the interface (see the orange dotted frame in Figure 6). In Figure 6b, vacancy defects appear on the first layer of the Fe matrix, resulting in the formation of a vacancy at the Fe2 position. Thus, the O1 and O2 can no longer bond with Fe2, prompting the O2 atom to relax toward the interior of the Al_2_O_3_, thereby reducing the bonding strength of the Fe/Al_2_O_3_ interface. It is worth noting that the vacancy in the third layer will induce the second layer of Fe atoms to move a large distance towards the vacancies, close to 1.2 Å, as shown by the arrows in Figure 6d. This results in the stretching of the chemical bonds between the first and second layers of Fe, concurrently with a slight shift of the first layer Fe atoms towards the Al_2_O_3_ side. In contrast to the ideal Fe/Al_2_O_3_ interface, the bond length of Fe-O in the V_3_ structure is shortened and the bond energy of Fe-O is increased, thereby enhancing the bonding strength of the interface. Therefore, the relaxation behavior of atoms provides a good explanation for the aforementioned calculation results.

In summary, when a vacancy defect appears in the first layer of the Fe matrix, it will have a greater negative impact on the bonding of the Fe/Al_2_O_3_ interface. Only when the vacancy defect appears in the third layer will the bonding strength of the interface be improved. To reveal the reason, we continue to make a deeper dissection of the interface structure from the electronic level.

### 3.2. Electronic Properties of Interface

In general, parameters such as electron density, electron density difference, overlap population of bonds, and density of states can provide insight into the chemical properties, binding strength, and bonding mechanism of the interface. Here, the electronic parameters of the ideal and non-ideal Fe/Al_2_O_3_ interface are calculated.

**Figure 7:**
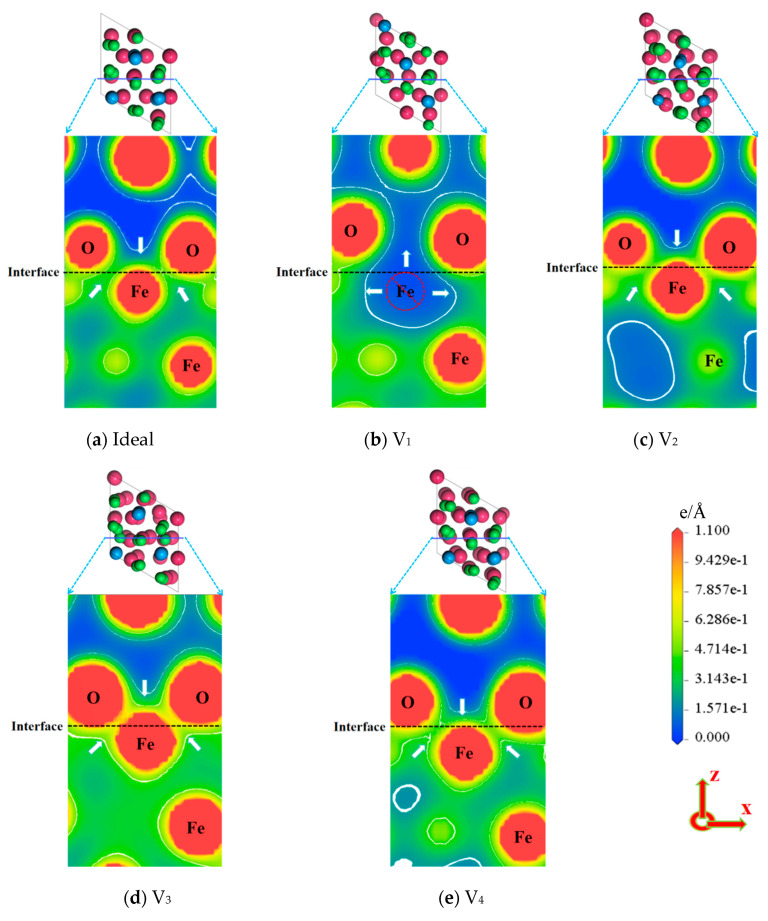
Charge density of ideal and defective Fe/Al_2_O_3_ interface structures.

Figure 7 shows the charge density at the ideal and non-ideal Fe/Al_2_O_3_ interface, where the change of color from blue via yellow to red represents a gradual increase in charge density from zero. It can be seen that all the Fe/Al_2_O_3_ interface regions show obvious charge aggregation (see the white arrow in Figure 7), which marks the occurrence of bonding and the formation of new substances at the interface. Compared to the ideal Fe/Al_2_O_3_ interface, the charge density of the V_3_ interface is significantly enhanced, which means that the bonding strength of the V_3_ interface is greater than that of the ideal Fe/Al_2_O_3_ interface [18]. However, due to the lack of Fe atoms of surface layer in the V_1_ structure, a charge depletion region appears at the interface in Figure 7b, which reduces the bonding strength of the interface. Additionally, the V_2_ and V_4_ structures have less influence on the interface compared to the ideal Fe/Al_2_O_3_ interface. The results show that the vacancy induces the rearrangement of electrons at the interface, and the increase of the charge concentration will improve the bonding strength of the Fe/Al_2_O_3_ interface, while the decrease of the charge concentration will reduce the bonding strength of the interface. This observation is consistent with the results reported by Yang et al. [30] regarding the charge density distribution at the RE(La,Ce)AlO_3_/Fe interface.

**Figure 8:**
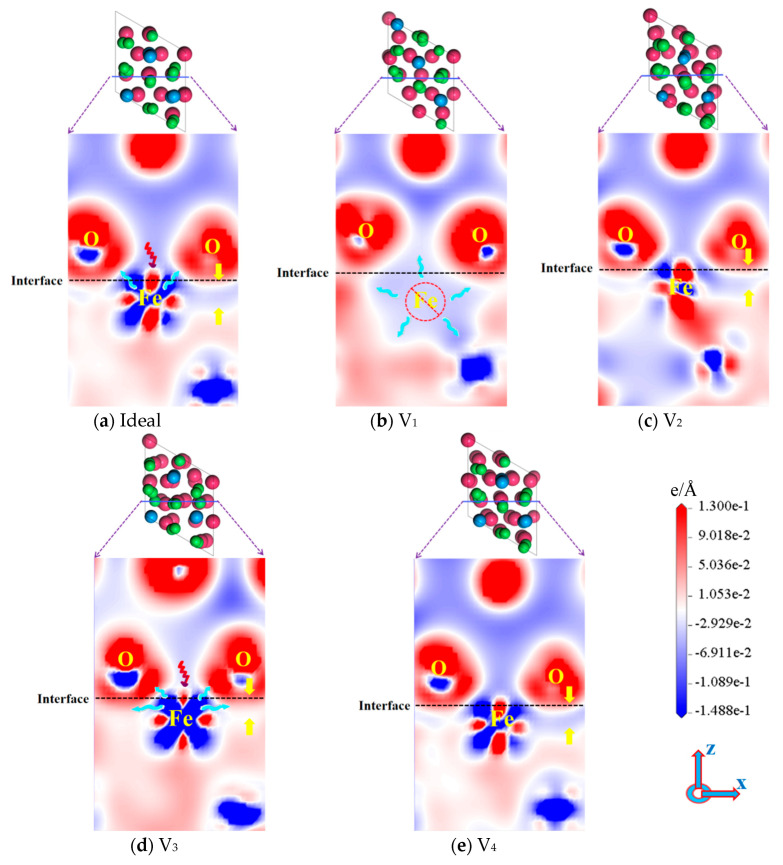
Charge density differences of ideal and defective Fe/Al_2_O_3_ interface structures.

The charge density difference maps of the ideal and defective Fe/Al_2_O_3_ interface structures are illustrated in Figure 8, in which blue represents the charge depletion region and red represents the charge accumulation region. For the ideal Fe/Al_2_O_3_ interface, there are both electron-accumulating and electron-dissipating regions around the Fe atom in Figure 8a, showing both symmetry and regularity [37]. Additionally, the phenomenon of local electron dissipation also appears around the O atom. This is characteristic of strong polar covalent bonds [30], suggesting that chemical bonds have been formed between Fe and O at the Fe/Al_2_O_3_ interface, which also supports the above inference. Interestingly, a similar situation occurs in the V_3_ structure, but the electron dissipation region of Fe is significantly larger than the ideal structure (the blue area is deeper), as shown in Figure 8d. Accordingly, it can be inferred that the Fe-O covalent bond formed at the V_3_ interface is stronger than the ideal Fe/Al_2_O_3_ interface. Moreover, local electron-dissipation regions for Fe and O at the V_1_ interface are smaller than the ideal structure, indicating that fewer electrons are transferred to the interface, i.e., weak covalent bonds are formed. It is worth noting that the electron dissipation regions of V_2_ and V_4_ interfaces are similar to those of the ideal interface (as indicated by the yellow arrows in Figure 8), suggesting that the bonding performance of the three kinds of interfaces is comparable, which is in good accordance with the results of W_ad_ above. It can be inferred that electron transfer may induce the formation of surface vacancies, thereby affecting the interfacial bonding properties. However, during the preparation of composite materials, environmental radiation can trigger electron movement. Relevant experiments [38] have reported that the extent of damage can be reduced by adjusting irradiation parameters—for example, by introducing protective gases to suppress the formation of iron vacancies at the interface. This approach can mitigate electron transfer behavior, optimizing the interfacial structure. Specifically, subsequent experimental work could involve controlling irradiation parameters to minimize damage and introducing protective gases to inhibit Fe vacancy formation at the interface, ultimately optimizing the interfacial structure and material properties.

**Figure 9:**
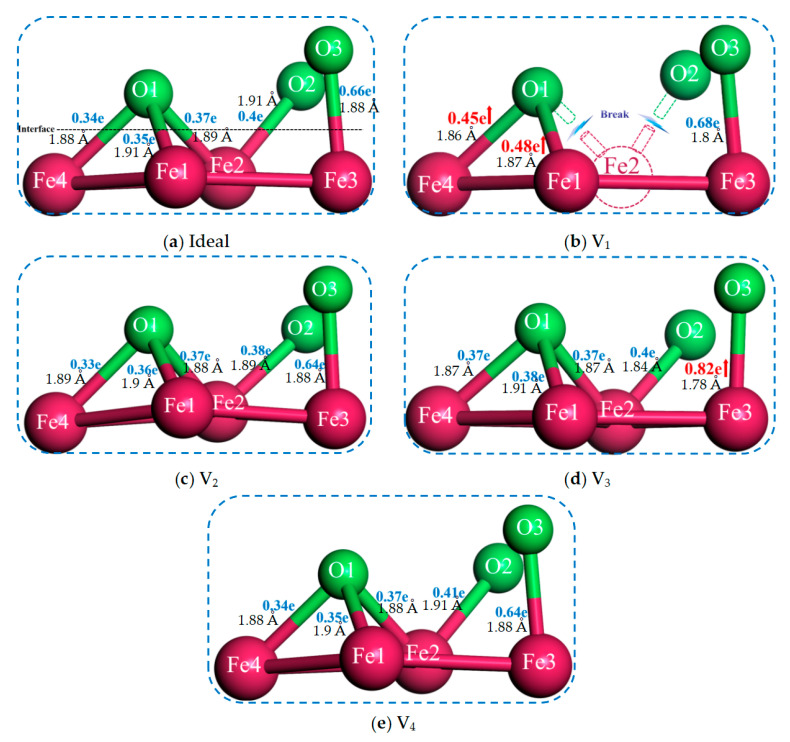
The overlap population of bonds for ideal and defective Fe/Al_2_O_3_ interface structures.

In order to further reveal the strength of the chemical bond at the interface, the overlap populations of the Fe-O bonds at the ideal and non-ideal Fe/Al_2_O_3_ interface are shown in Figure 9, where positive values of overlap population represent covalent characteristics. The larger the value of the overlap population, the stronger the chemical bond. Here, we count the sum of the overlap population of Fe-O bonds at each Fe/Al_2_O_3_ interface, in descending order: V_3_ (2.34 e), V_ideal_ (2.12 e), V_4_ (2.11 e), V_2_ (2.08 e), and V_1_ (1.61 e). The sequence of overlap population values matches precisely with the order of W_ad_ described above, indicating a direct relationship between the overlap population of chemical bonds and the binding strength at the Fe/Al_2_O_3_ interface. From the strength of the chemical bond aspect, it is also suggested that the V_3_ interface has the strongest bonding and the V_1_ is the weakest. To note, the overlap population of the Fe2–O2 bond in the V_3_ interface increased significantly (see the red arrow in Figure 9d), which increased by 24.2% compared to the ideal interface. This enhancement is further supported by a 5.3% shortening of the Fe2–O2 bond length relative to the other interfaces. Indeed, shorter chemical bonds typically correspond to higher bond energies, which further supports the conclusion that the V_3_ structure is the most stable. This reveals that the chemical bond strength is the key factor in determining the binding strength of an interface.

**Figure 10:**
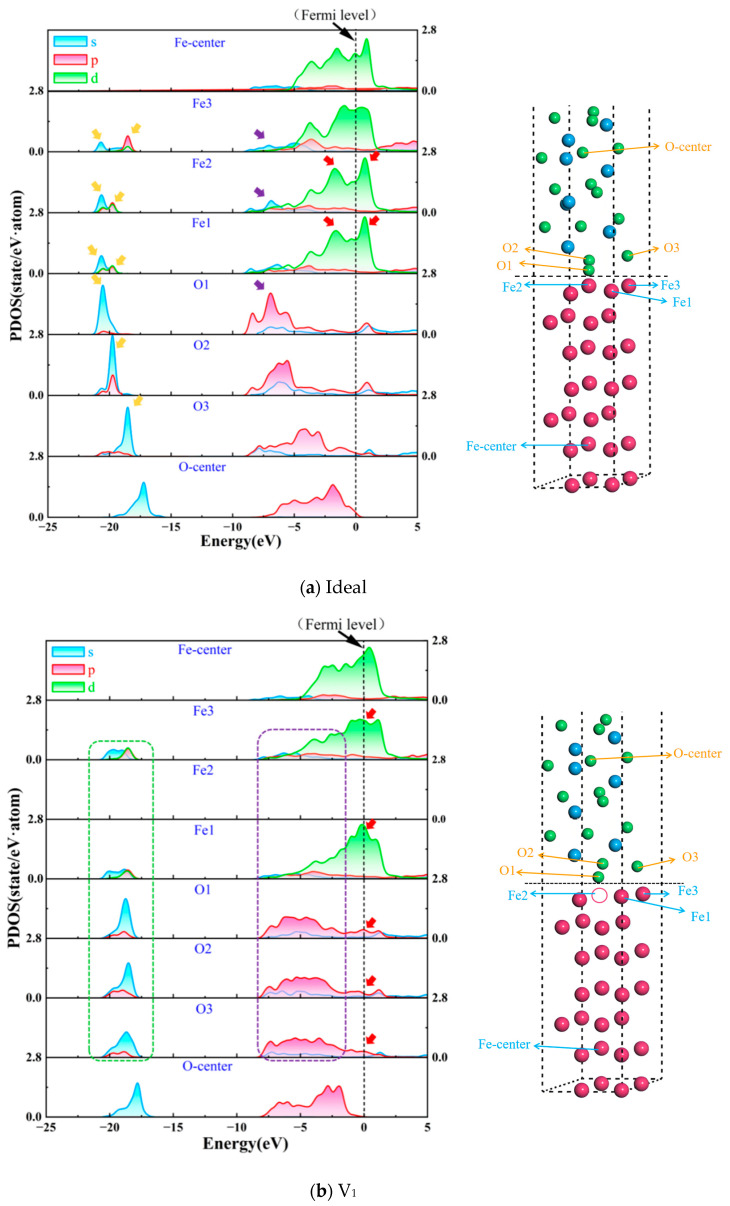

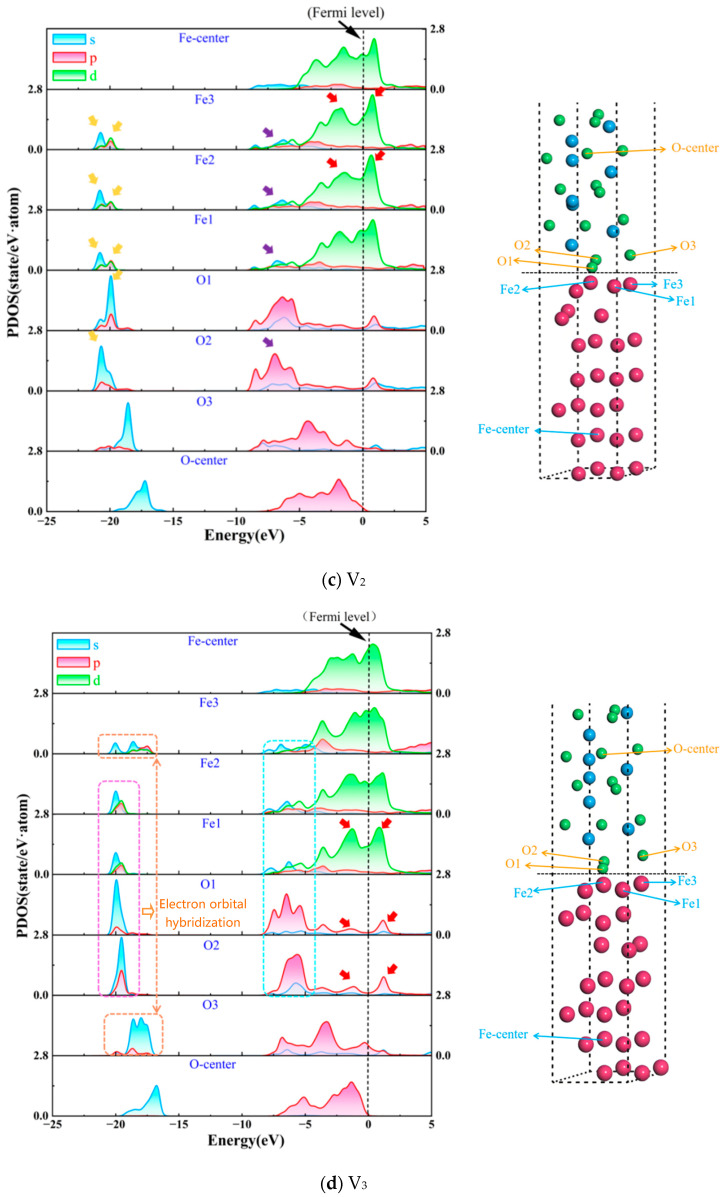

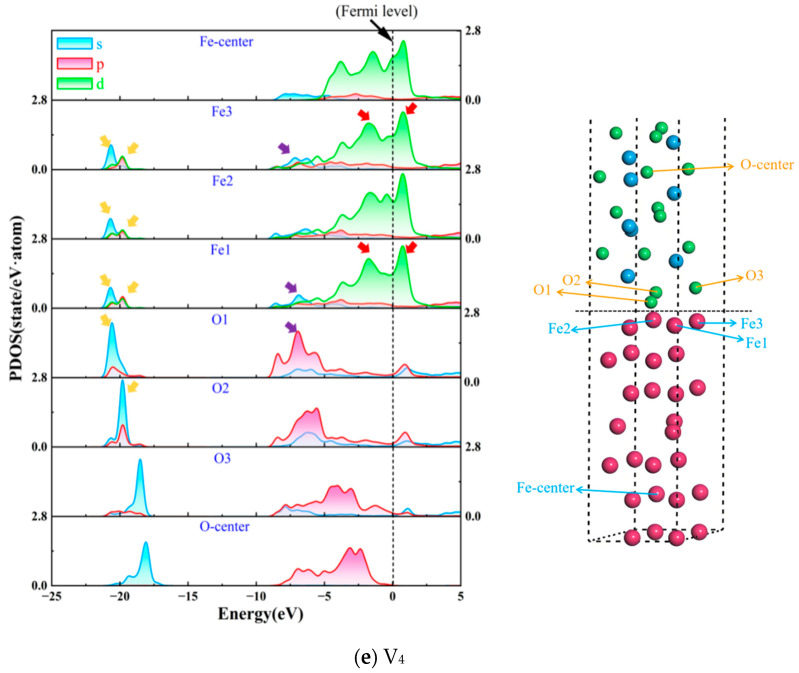
The PDOS of ideal and defective Fe/Al_2_O_3_ interface structures.

Indeed, the transfer of electrons at the interface will result in a change in the electron orbitals of the atom. As a result, it is necessary to calculate the partial density of states (PDOS) of the atoms at the interface to reveal the effect of the vacancy defect on the chemical properties of the Fe/Al_2_O_3_ interface. Figure 10 shows the PDOS of local atoms at ideal and non-ideal Fe/Al_2_O_3_ interfaces, where the black dotted line represents the Fermi level. For all structures, the PDOS peak of O atoms at the interface are prominent at the position of Fermi level, i.e., greater than zero. This means that the metallic bonds have been formed at all Fe/Al_2_O_3_ interface. Compared to the O-center and Fe-center atoms, the PDOS curves of O and Fe atoms change significantly, indicating that orbital hybridization of electron between O and Fe occur at the interface. For the V_1_ interface, the PDOS peak of the Fe and O atoms decreases obviously in the range of −21.63 to −17.62 eV (see green dotted box in Figure 10b) compared to the ideal Fe/Al_2_O_3_ interface, indicating that the orbital hybridization effect is weakening. In Figure 10d, the Fe3 and O3 in the V_3_ interface form an orbital hybridization of electrons significantly stronger than the ideal interface from −21.9 to −16.1 eV, presenting stronger covalent properties. Of further note, the orbitals between O-2p and Fe-3d form a clear double-hump on either side of the Fermi level, displaying typical pseudogap characteristics [39], alluding to the formation of significant electron-sharing regions at the interface. Actually, this is the root cause of the variation in the bonding strength of Fe/Al_2_O_3_ interfaces caused by vacancy defects. Moreover, the PDOS curves of V_2_ and V_4_ (Figure 10c,e) are not significantly different compared to the ideal interface, which also indicates that vacancies at these positions have little effect on the interface.

**Figure 11:**
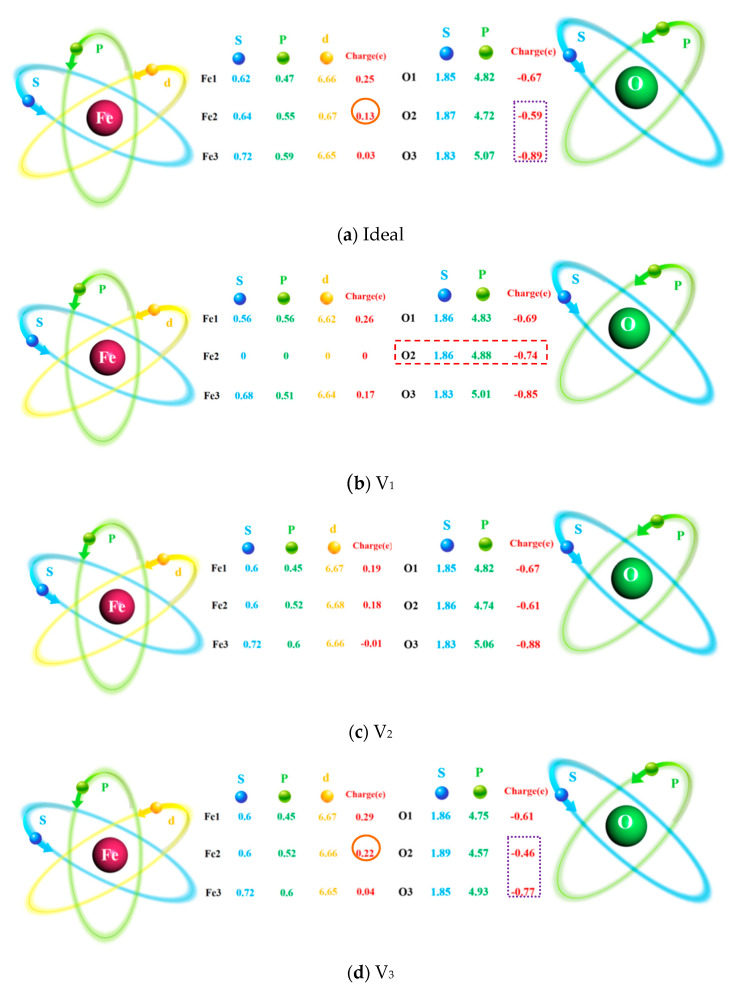

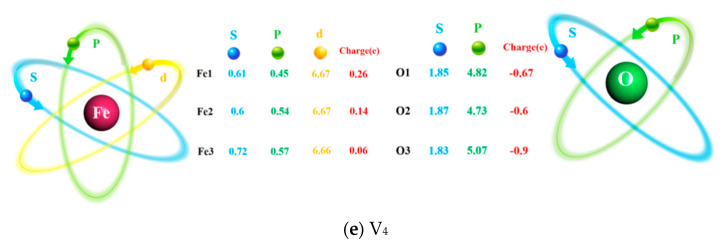
Atomic populations of ideal and defective Fe/Al_2_O_3_ interface structures.

To further explore the changing behavior of the electronic orbits at the Fe/Al_2_O_3_ interface, the Mulliken populations [40] of the atoms are calculated as shown in Figure 11. It is found that the gain and loss of orbital charges in the V_2_ and V_4_ interface is negligible compared to the ideal Fe/Al_2_O_3_ interface. Interestingly, the extra-nuclear charge of O2 in the V_1_ interface increases from −0.59 to −0.74 e compared to the ideal interface. Owing to the fact that orbital electrons at the interface do not change significantly, it can be inferred that the vacancy defect of the outermost layer of the Fe surface is leading in the transfer of electrons from the interface to the O2 atom. As a result, the charge concentration of the V_1_ interface decreases, resulting in a reduced binding strength of the Fe/Al_2_O_3_ interface. In the V_3_ structure, the extra-nuclear charge of Fe2 is decreased by 69.2% compared to the ideal structure, while the O2 and O3 charges decrease by 18.1%. This is mainly caused by more orbital hybridization of electrons between Fe and O, showing strong covalent characteristics.

Overall, the impact of Fe vacancy defects on the Fe/Al_2_O_3_ interface can be divided into four steps. First, vacancies induce the relaxation behavior of atoms at the interface. Second, chemical bonds between atoms break and reform. Third, electrons at the interface undergo transfer and rearrangement. Fourth, the hybridization effect of the new electron orbits occurs. These processes will directly alter the bonding mechanism and binding strength of the Fe/Al_2_O_3_ interface.

## 4. Conclusions

In this paper, a fixed vacancy concentration of 25% was maintained while only the vacancy positions were modified. The V1, V2, V3, and V4 configurations were constructed and compared with the ideal interface, with particular focus on the influence of vacancy location on interfacial bonding properties. The specific conclusions are as follows:

(1) In contrast to the ideal Fe/Al_2_O_3_ interface, the bonding strength of the V_1_ interface decreased by about 12%, while the bonding strength of the V_3_ interface increased by nearly 18%. Vacancies progressively change from bond-weakening to bond-strengthening as they move inward from the first to third atomic layers, becoming ineffective beyond this depth. Given the importance of reducing vacancy defects in the first layer of the Fe matrix for improving the interfacial bonding strength of Fe-Al_2_O_3_ composites, several strategies could be explored in future studies. These might include controlling irradiation-induced damage during experiments. The introduction of a protective atmosphere, such as helium, may also be beneficial to suppress vacancy formation and thereby optimize interfacial performance.
(2) The existence of vacancies at various positions will induce the relaxation behavior of atoms at the interface, which creates the bond-breaking and bond-forming reactions between atoms at the Fe/Al_2_O_3_ interface. This alters the environment of the interface region and directly affects the interfacial bonding performance.
(3) Vacancy defects at the Fe/Al_2_O_3_ interface promote electronic redistribution and enhance interatomic orbital hybridization, resulting in a charge-sharing region. The charge density in this region varies with the vacancy type, being lowest in the V1 structure and highest in V3. In other words, the higher the charge concentration at the interface, the stronger the binding strength at the interface. This charge concentration shows a positive correlation with the interfacial bonding strength. Furthermore, increased orbital hybridization strengthens the interfacial bonding, which fundamentally explains how vacancy defects modulate the bonding properties of the interface.

## Data Availability

The original contributions presented in this study are included in the article. Further inquiries can be directed to the corresponding author.
